# Using the GRADE‐ADOLOPMENT framework in developing the Philippine national screening guideline recommendations

**DOI:** 10.1002/gin2.70008

**Published:** 2024-12-17

**Authors:** Ian Theodore G. Cabaluna, Maria Vanessa C. Villarruz‐Sulit, Katelyn Edelwina Y. Legaspi, Leonila F. Dans

**Affiliations:** ^1^ Institute of Clinical Epidemiology, National Institutes of Health University of the Philippines Quezon City Philippines; ^2^ Department of Clinical Epidemiology, College of Medicine University of the Philippines Quezon City Philippines; ^3^ Department of Pediatrics, College of Medicine University of the Philippines Quezon City Philippines

**Keywords:** adolopment, LMIC, Philippines, preventive screening guidelines

## Abstract

**Background:**

Preventive health screening is an important strategy in improving health outcomes of the general population and sustaining universal healthcare initiatives. Due to the large resource requirement, we used the GRADE ADOLOPMENT framework to synthesize the evidence and develop recommendations for preventive health screening.

**Objective:**

Our objectives were to describe the development and feasibility of a national preventive screening practice guideline using the GRADE‐adolopment process and to discuss the methodological process, contextual differences from the source guidelines, and the resulting changes to the final recommendations.

**Methods:**

A multidisciplinary team was convened. We used the GRADE‐ADOLOPMENT and Evidence‐to‐Decision (EtD) framework in synthesizing the evidence and developing the recommendations. Evidence from the World Health Organization, United States Preventive Services Task Force, and Canadian Task Force on Preventive Healthcare were used. Values and preferences of the guideline developers were incorporated through the EtD framework.

**Result:**

In 6 months, we developed 16 evidence summaries and developed 23 recommendations for 16 prioritized conditions. Thirteen recommendations were adopted. Four recommendations were modified to address contextual differences. Six recommendations were developed de novo due to either lack of evidence or differences in values and preferences.

**Conclusion:**

The GRADE‐adolopment framework was a feasible and efficient framework in adopting guidelines on preventive screening. The EtD framework improved the transparency and highlighted areas to consider in making recommendations for the Philippine context. Challenges encountered were insufficiency of local evidence, and the lack of experience and skills in interpreting and analyzing the evidence especially on screening strategies.

## Highlights

- In 6 months, the Philippine Guideline Periodic Health Examination (PHEX) working group developed 16 evidence summaries and developed 23 recommendations for 16 prioritized conditions using the GRADE‐ADOLOPMENT framework.
- The GRADE‐adolopment framework proved feasible and efficient for adapting preventive screening guidelines for the Philippines.
- Challenges included lack of contextual evidence, and limited expertize and experience in adapting and synthesizing screening evidence.

## 1 INTRODUCTION

Preventive disease screening is a common strategy that uses early detection and management to improve quality of life and prevent premature death. Ideally, this would lead to early treatments that are less invasive and more cost‐effective than those that would be required at a later stage of the disease.^1^

Many illnesses may be over‐ or under‐diagnosed in patients. Some screening findings ultimately do not turn into clinical symptoms or affect the patient's lifespan or quality.^2^ Overdiagnosis can result in invasive testing, excessive treatment, anxiety, stress, and stigma for the patient without a commensurate benefit in health outcomes.^1^ On the other hand, underdiagnosis could lead to a delay in disease detection thus allowing it to progress, false reassurance to the patient, and loss of faith in the medical profession.^3^

The benefits and harms of screening tests are not always clear, especially when there is a lack of high‐quality data regarding that specific screening test and disease outcome. Trustworthy clinical practice guidelines (CPGs) are meant to summarize current information regarding particular health conditions for the benefit of physicians, patients, and health systems alike. It aids in maximizing the benefit and minimizing the harm of such interventions.^4^ They can promote interventions with proven benefit, and discourage ineffective practices.^4^, ^5^

To support the country's push for universal health, the Philippine Department of Health partnered with the Institute of Clinical Epidemiology (ICE), National Institutes of Health (NIH) of the University of the Philippines to update the 2004 Philippine Periodic Health Examination (PHEX) Guidelines and the creation of the PhilHealth Konsultasyong Sulit Tama (Konsulta), a comprehensive outpatient benefits package as mandated by the Universal Health Care Law.^6^, ^7^, ^8^

The guidelines were urgently needed, but creating them from scratch would have been impractical due to the time, effort, and resources required. To overcome these challenges, the task force used the GRADE adolopment framework. This process enables guideline makers to adapt existing guidelines to their own contexts and capabilities rather than starting from scratch. The framework was used in formulating the PHEX guidelines also because it allows incorporation of the GRADE approach and gives an explicit and simple process in adapting guidelines. The term “adolopment” was coined by the GRADE Working Group as a structured approach to combining the advantages of selectively combining adoption, adaptation, and de novo development of guideline recommendations.^9^ GRADE defines adoption as contextualization without changes to the original recommendation from source guidelines, while adaptation involves using the original recommendations and contextualizing them given local or regional evidence with possible changes to the recommendation or the EtD.^10^

The PHEX project intended to update the screening guidelines in three phases. Phase 1 had the objective of developing recommendations for screening interventions already in use. These screening interventions were already included in the government outpatient package but have not been thoroughly reviewed and recommended in a local CPG. Phase 1 was intended to serve as a pilot project to which phase 2 and 3 will be grounded on. The following phases 2 and 3 are meant to evaluate screening strategies that are considered to be novel, controversial, or misused, along with the creation of an online mobile app that would make it easier for clinicians to apply the results of this evidence review to their individual patients. In this paper, we described our experience in phase 1 (Supporting Information S1).^11^

This article also describes the methodologic process in the development of the Philippine Periodic Health Examination Guidelines 2021 through the use of the GRADE‐ADOLOPMENT process.^12^ The methodological process, contextual differences between the source guidelines and the Philippines will be discussed, as well as the resulting changes to the final recommendations.

## 2 METHODS

### 2.1 Recruitment of team

Three groups were created to carry out this project. These were the steering committee, technical working group (TWG) and the consensus panel. The teams had different roles and functions. See Table 1.

**Table 1:** Roles of the task force members.

Position and number recruited	Composition	Role description
Steering committee (SC)	Experts with diverse experience in the field of primary health care and public health, evidence‐based medicine and guideline development	Supervision of the guideline development process Determination of scope and guideline questions
Technical working group (TWG)	Experts with previous knowledge of evidence‐based medicine and evidence synthesis	Develop and present the evidence
Consensus panel (CP)	Representatives from the government, private physicians, occupational physicians, public health physicians, nurses, and patients.	Develop and finalized the recommendation

The steering committee were composed of individuals from multiple disciplines that would be affected by the guidelines and had experience, knowledge and skills in evidence‐based medicine and guideline development. The group oversaw the whole development. The TWG were evidence review experts, all with training in the analysis of data and evidence‐based medicine. They performed systematic searches, appraisal and synthesis of evidence. A technical coordinator supervised the TWG in the synthesis and presentation of evidence. The consensus panel was a multi‐disciplinary group composed of 12 members representing varied stakeholders. There were representatives for the government, private physicians, occupational and public health physicians, nurses, and patients. The consensus panel developed recommendations based on the evidence presented and the values and preference of their represented sector.

#### 2.1.1 Conflict of interest

All members of the Steering Committee, TWG and nominees to the Consensus Panel were required to submit their curriculum vitae and to declare their potential conflict of interest using a declaration of COI form. The Steering Committee screened and reviewed for potential COI of the Consensus Panel nominees. Only those without potential COIs were allowed to vote.

#### 2.1.2 Prioritization of topics/questions

Screening interventions included in the country's primary care outpatient package, Konsulta Package, and screening interventions for topics deemed by the Screening Committee to be a priority were included. All the diseases being screened for were deemed to have high disease burden in the Philippines. Conditions and risk factors deemed to have considerable health impact on society and with high variability in practice were considered in prioritizing the diseases/risk factors to screen. Other conditions that were not prioritized in this phase were covered in the next phases of the PHEX guidelines.

The topics selected for the first phase of the PHEX guidelines can be divided into two categories: screening for early disease and screening for risk factors for disease development. Screening for early disease allows for the early detection and treatment of the disease, which could potentially stop it from progressing further. Screening for risk factors provides the opportunity to prevent the disease process from even starting.

After the topics were chosen, the guideline questions and accompanying research questions in PICO format were developed. In all the questions, the population was asymptomatic, apparently healthy individuals, while the interventions were screening tests that the Philippine government was currently covering for the prioritized topics. The SC determined the initial outcomes but were subsequently rated by the CP. Patient‐important outcomes such as mortality and morbidity were considered.

#### 2.1.3 Evidence synthesis

The ADOLOPMENT approach were done through the following steps.^13^

**Figure 1:**
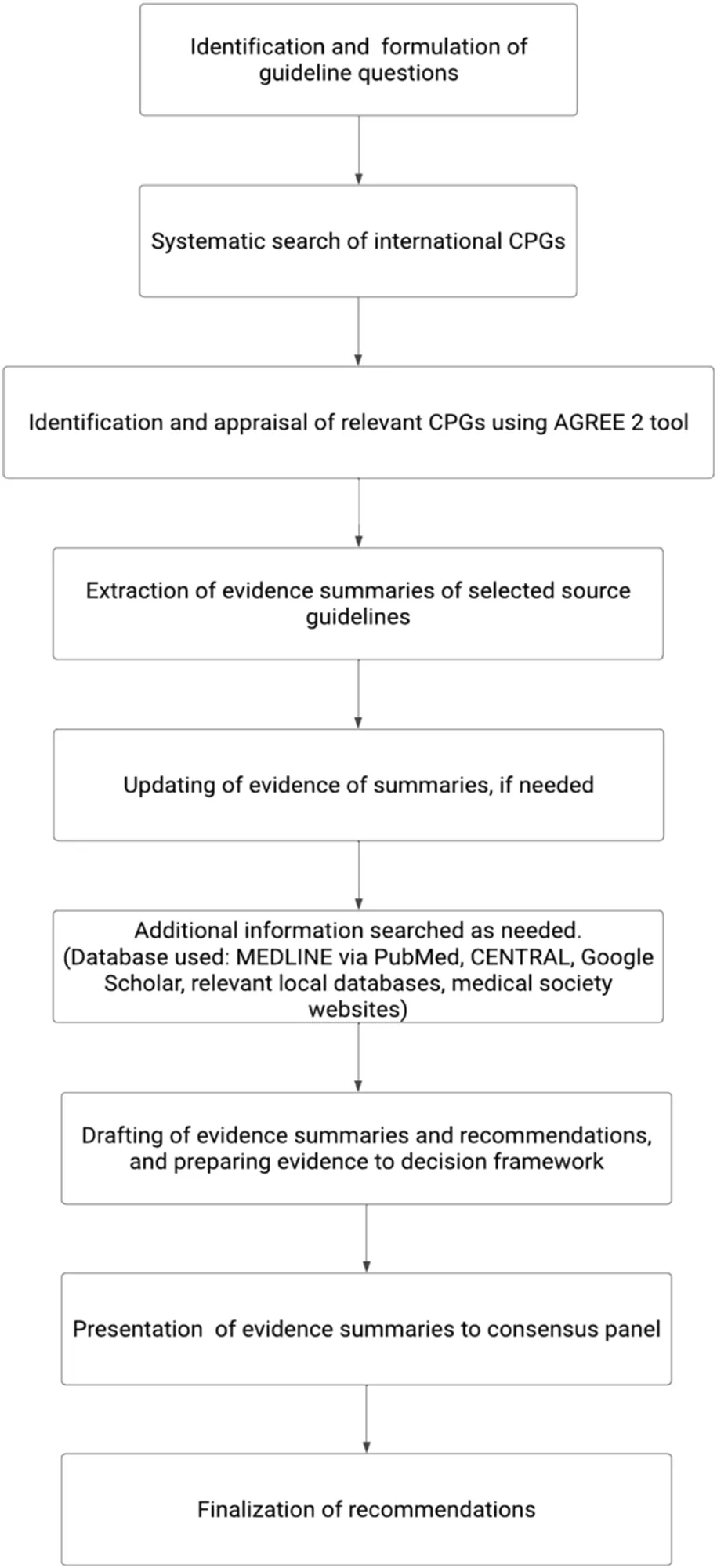
Methodology used in the development of PHEX.

1. Evidence from source guidelines were gathered and synthesized for each chosen topic, their relative strengths and importance were assessed through the GRADE approach.
2. The evidence was used to fill in the GRADE EtD frameworks for each recommendation. Additional local and contextual evidence were searched to supplement the EtD.
3. The GRADE EtD frameworks together with the evidence summaries were presented to the consensus panel, wherein they would decide on the direction and strength of the recommendations. See Figure 1 for details.

Guidelines were found by performing a systematic search of resources published by various international guideline‐developing bodies websites and through PubMed. Recurring organizations include the World Health Organization, the United States Prevention Task Force, the Canadian Task Force on Preventive Healthcare, and the National Institute for Health Care and Excellence.

Two independent reviewers appraised the guidelines found using the AGREE II tool.^14^, ^15^ These were accepted if they were published after 2015, and were deemed to be of high quality (AGREE II score >75%). When multiple guidelines answering the same question were found, guidelines with the most recent evidence were used. The decision to adapt, adopt, or create de novo occurred after gathering the evidence from source guidelines.

After the guidelines were chosen, the TWG extracted and updated the evidence presented by the source guidelines. If the source guideline's evidence last search was more than a year, a search was done to check for new evidence. Evidence included the burden of the problem, the diagnostic performance, balance between benefits and harms of screening, and social and economic impact of the proposed screening intervention. When no available evidence was found from the source documents, a de novo systematic search and evidence synthesis was performed. Meta‐analysis was performed for important clinical outcomes if appropriate. Except for the WHO guidelines on TB, the source guidelines used did not provide an EtD. At the onset, the steering committee has decided to create the EtD by using applicable evidence from the source guideline. Specifically, evidence on benefit and harms of screening, diagnostic accuracy, and certainty of evidence were used. Additional evidence such as applicability or feasibility for the EtD was obtained using a systematic search process looking at local publications through national surveys and different publications of medical societies as well as using ancestry searching.

All evidence summaries developed for use in these guidelines have been recommended for publication. A technical coordinator with expertize on CPG development and Evidence‐based medicine oversaw the retrieval and appraisal of evidence, and the creation of the draft recommendations. Consultations and practice online presentations with the project leader, manager, technical coordinator and steering committee were held as needed before the consensus panel meetings. The finalized evidence summaries were sent to the consensus panel before the meetings.

#### 2.1.4 Evidence to decision

The consensus panel was oriented on how to interpret the evidence summaries, the EtD framework used to synthesize the evidence, and the voting process that would follow to reach the final recommendation, before the actual consensus meeting. Before the meeting, they were also required to answer the following questions based on the EtD criteria and required judgements.

1. Should (screening topic) be a priority?
2. How substantial are the desirable anticipated effects?
3. How substantial are the undesirable anticipated effects?
4. Does the balance between desirable and undesirable health effects favor screening or no screening?
5. How accurate is the test or strategy?
6. How certain is the link between tests results and management decisions? (How likely will the management be carried out given the test result?)
7. How large are the resource requirements (costs)?
8. Does the cost‐effectiveness of the test favor the test or the comparison?
9. Does the screening intervention improves or worsen equity?
10. Is the screening intervention acceptable to the stakeholders?
11. Is the screening intervention feasible to the stakeholders?

These were summarized and presented during the actual consensus meeting.

During the meeting, a facilitator moderated the discussion. The evidence to decision framework was used to present the data to the consensus panel. Since the recommendations were about diagnostic and screening tests, the consensus panel was asked to consider outcomes important to patients, such as mortality and the burden of subsequent interventions. The balance of benefit versus harm, diagnostic accuracy, certainty of evidence, cost, acceptability, feasibility, appropriateness, and equity was also considered.

Time was given for clarification and discussion after each presentation of evidence, the panel would move to vote on the direction of the recommendation. If consensus (75% of the total votes) was reached on or before the third round, the panel would then proceed to vote on the strength of the recommendation. When there was a screening intervention with no evidence, the E‐Delphi process was used to arrive at a consensus. The consensus panel reached consensus in recommendations for all 16 guide questions. Recommendations from the source guideline and other relevant guidelines were also presented but were not deliberately discussed during the panel meeting to prevent bias towards the source guidelines' recommendations. Instead, the panel decided on the direction and strength of the recommendations based on the EtD.

## 3 RESULTS

### 3.1 Timeframe

It took 6 months to develop the 23 recommendations for screening 16 prioritized conditions. Two months were required for guideline preparation, 3 months were used to produce the evidence summaries, and the final month consisted of finalizing the recommendations generated by the team.

### 3.2 Source guidelines

The primary source guidelines included had an AGREE II score of 100 for rigor of development. These were the United States Preventive Services Task Force (USPTF), the Canadian Task Force on Preventive Health Care (CTFPH) and the World Health Organization consolidated guidelines on tuberculosis.

Clinical outcomes deemed critical by the PHEX team matched those of the source guidelines and became a major basis of the decision. The decisions were also supplemented by local evidence on burden of the disease, acceptability, cost effectiveness and feasibility, when available. Although the primary source guideline and the Philippines had different contexts and considerations, the PHEX team ultimately chose to give similar recommendations for 13 topics. See Table 2.

**Table 2:** Summary of PHEX recommendations and action taken.

PHEX 2020 recommendations	Source guideline	Adopted, adapted, or de novo	Considerations	Additional work required
Among asymptomatic, apparently healthy adults aged 40–75 years with one or more CV risk factors, we suggest screening for lipid disorder using a lipid profile test. (weak recommendation, low certainty evidence).	USPSTF^16^	Adopted	The USPSTF guideline did not specifically focus on lipid screening but on whether to start taking statins. The recommendation on lipid screening was based on the fact that lipid profile testing is necessary to determine the 10‐year cardiovascular risk.	Local evidence was searched for the EtD.
Among asymptomatic, apparently healthy adults aged 18–39 years, we suggest against screening for lipid disorder (weak recommendation, no evidence).	USPSTF^16^	Adapted		Local evidence was searched for the EtD.
Among asymptomatic apparently healthy adults, we recommend screening for hypertension (strong recommendation, moderate certainty evidence).	USPSTF^17^	Adopted	No significant difference.	Local evidence was searched for the EtD.
Among apparently healthy adults aged 40 years and above, or younger if with risk factors, we recommend screening for type 2 diabetes mellitus using fasting blood sugar (strong recommendation, moderate certainty evidence).	USPSTF^18^	Adapted	Due to contextual circumstances in the Philippines, PHEX provided only a conditional recommendation for HBA1c. This test is not readily available in the country and has yet to be standardized between testing centers. Different laboratories may provide various ranges of reference values.	Local evidence was searched for the EtD.
Amongst apparently healthy adults aged 40 years old and above, or younger if risk factors*, we suggest screening for type 2 diabetes mellitus using hemoglobin A1c (conditional recommendation, moderate certainty evidence).	USPSTF^18^	Adapted		Local evidence was searched for the EtD.
Among asymptomatic apparently healthy adults, we recommend using body mass index in screening for obesity (strong recommendation, moderate certainty evidence).	CTFPHC^19^, ^20^	Adopted	CTFPHC focused on overnutrition and obesity in both children and adults. There was not much discussion of undernutrition or malnutrition, both of which are still prevalent problems in the Philippines. PHEX panel decided to use the Asia‐Pacific Classification for cut‐off scores on BMI as that was deemed more appropriate to the local setting.	Local evidence was searched for the EtD.
Among asymptomatic apparently healthy children (6–59 months), we suggest screening for acute malnutrition using mid‐upper arm circumference or weight‐for‐height z‐scores. (conditional recommendation, low certainty evidence).	CTFPHC^19^, ^20^	Adopted		Local evidence was searched for the EtD.
Among asymptomatic apparently healthy nonpregnant adults, we suggest against routine screening of anemia using hemoglobin and/or hematocrit (conditional recommendation, very low certainty evidence).	N/A	De‐novo	No source CPGs were identified.	Systematic reviews and meta‐analyses on the effectiveness and harm of screening, diagnostic accuracy, and treatment were done.
Among asymptomatic apparently healthy adults, we suggest against screening for chronic kidney disease using urinalysis (conditional recommendation, low certainty evidence).	N/A	De‐novo	No source CPGs were identified.	Systematic reviews and meta‐analyses on the effectiveness and harm of screening, diagnostic accuracy, and treatment were done.
Among asymptomatic apparently healthy adults, we recommend against screening for chronic kidney disease using serum creatinine (strong recommendation, no evidence).	N/A	De‐novo	No source CPGs were identified.	Systematic reviews and meta‐analyses on the effectiveness and harm of screening, diagnostic accuracy, and treatment were done.
Among asymptomatic apparently healthy adults, we suggest against screening for lung cancer using a chest x‐ray (conditional recommendation, low certainty evidence).	USPSTF^21^	Adopted	No significant difference.	Local evidence was searched for the EtD.
Among asymptomatic apparently healthy adults, we suggest screening for pulmonary tuberculosis using a chest x‐ray (conditional recommendation, very low certainty evidence).	WHO Consolidated guidelines on tuberculosis^22^	Adopted	No significant difference.	Local evidence was searched for the EtD.
Among asymptomatic apparently healthy adults, we recommend against the use of resting or exercise ECG to screen for coronary artery disease (strong recommendation, low certainty evidence).	USPSTF^23^	Adopted	No significant difference.	Local evidence was searched for the EtD. Updating of source guideline was done.
Among asymptomatic women aged 21–65, we recommend screening for cervical cancer every three years using a Pap smear (strong recommendation, moderate certainty evidence).	USPSTF^24^	Adopted	The USPSTF recommends cervical cytology and HPV testing. Visual inspection with acetic acid (VIA) is not mentioned. However, the PHEX panel still decided to recommend visual inspection with acetic acid as an alternative to pap smear despite the moderate certainty of evidence due to issues of inequity and accessibility.	Local evidence was searched for the EtD.
Among asymptomatic women aged 21–65, we recommend screening for cervical cancer every three years using visual inspection with acetic acid as an alternative to Pap smear (strong recommendation, moderate certainty evidence).	N/A	De‐novo		Local evidence was searched for the EtD.
Among asymptomatic apparently healthy adults aged at least 50, we recommend to screen for colorectal cancer using annual FOBT or FIT, followed by colonoscopy, when indicated. (strong recommendation, high certainty evidence).	USPSTF^25^	Adapted	The USPSTF guideline recommends tests that are not yet widely available or affordable in the Philippines. In addition, the USPSTF recommends colonoscopy at regular intervals, while for PHEX, it is only performed when indicated. This is due to the prohibitively high cost of colonoscopy in the Philippines. Although many practitioners are trained to perform it, its accessibility is limited, especially in rural areas. Additionally, the age at initiation and termination of screening is different for the two guidelines owing to local prevalence of the disease.	Local evidence was searched for the EtD.
Among asymptomatic apparently healthy adults, we recommend screening for unhealthy alcohol use and providing persons identified with risky drinking with brief behavioral counseling intervention. (strong recommendation, moderate certainty evidence).	USPSTF^26^	Adopted	No significant difference.	Local evidence was searched for the EtD.
Among asymptomatic apparently healthy adolescents, we suggest screening for unhealthy alcohol use and providing persons identified with risky drinking with brief behavioral counseling intervention (conditional recommendation, low certainty evidence).	N/A	De‐novo	PHEX decided to give an outright recommendation regarding screening despite the lack of evidence in adolescents.	Local evidence was searched for the EtD.
Among asymptomatic apparently healthy adolescents and adults, we suggest screening for high‐risk sexual behavior (conditional recommendation, low certainty evidence).	N/A	De‐novo	No source CPGs were identified.	Systematic reviews and meta‐analyses on the effectiveness and harm of screening, diagnostic accuracy, and treatment were done.
Among all adults, we recommend that healthcare providers screen for tobacco smoking (strong recommendation, low certainty evidence).	USPSTF^27^	Adopted	However, the PHEX guideline ranked the evidence as only low certainty. The difference in the recommendation stems from the fact that the PHEX panel limited itself to screening for tobacco smoking, but the USPSTF already includes interventions within its guidelines.	Local evidence was searched for the EtD.
Among all adolescents, we recommend that healthcare providers screen for tobacco smoking (strong recommendation, very low certainty evidence).	USPSTF^27^	Adopted		
Among asymptomatic apparently healthy adolescents and adults, we suggest screening for depression using PHQ‐9 (conditional recommendation, low certainty evidence).	USPSTF^28^, ^29^	Adopted	No significant changes	Local evidence was searched for the EtD.
Among asymptomatic apparently healthy children, we recommend against screening of developmental delay using developmental delay screening tools (strong recommendation, low certainty evidence).	CTFPH^30^	Adopted	No significant changes	Local evidence was searched for the EtD.

### 3.3 Recommendations

Four recommendations were adapted with changes to account for context‐specific circumstances. Due to the insufficiency of the evidence, the panel decided to conditionally recommend against screening for lipid disorders among those less than 40 years old instead of having no recommendation. The panel decided that the recommendation by the source guideline for diabetes mellitus could not be recommended as is.^14^ The panel decided to provide only a weak recommendation for using HBA1c as a diabetes screening tool because the test is not readily available in all regions of the Philippines and its calibration is not standardized nationwide.

Four recommendations were made de novo. De‐novo recommendations for screening unhealthy alcohol use in adolescents were made despite the low certainty of evidence because alcohol misuse was deemed to be a priority risk factor to screen. Similarly, the guideline for screening against chronic kidney disease in healthy adults was made because of the insufficient evidence of its benefit. No guidelines were identified that explicitly addressed the issue of screening for high‐risk sexual behaviors in apparently healthy, asymptomatic adolescents. The panel raised the issues of privacy, confidentiality, stigma, and the cost of potentially recommended laboratory tests. However, the recommendation was still pushed forward as a weak recommendation due to the rising number of cases of HIV in the Philippines. An additional recommendation on the use of VIA for screening cervical cancer was made de novo. This was because of perceived inequity and poor accessibility to pap smear in the Philippines.^11^

## 4 DISCUSSION

To the best of our knowledge, this was the first in the Philippines that the GRADE‐ADOLOPMENT framework was used to develop CPG recommendations. We found the GRADE‐ADOLOPMENT framework feasible in adapting guidelines in countries with minimal resources. It allows fast‐turn out of recommendations and still considers important local context.

Using the GRADE ADOLOPMENT framework, it took us 6 months to develop the recommendations including preparation and evidence synthesis. This is a similar time frame to other guideline developers that ranges from 4 months to 1 year.^31^, ^32^, ^33^

This experience proves that using the framework may decrease the time it takes to come up with recommendations. Searching for primary evidence was cut short and allows to focus on searching for local evidence needed to make a localized recommendation. It also does not diminish the thorough process required in guideline development but allows for a merging of strategies in obtaining evidence needed for a question. It also acknowledges that every setting is different and specific concerns are carefully considered. Therefore, the benefit comes in its efficiency in the way the recommendations are arrived at. It is in being efficient that resources are maximized.

The recommendations were modified based on the disease burden, feasibility, and economic impact. Some questions lacked existing recommendations, or the source guideline did not provide a clear recommendation due to insufficient evidence. However, despite the lack of evidence, the panel decided to create recommendations for diseases with a high burden in the Philippines. The panel recognized the importance of these recommendations to address the impact of these diseases. The availability and accessibility of tests across the country also modified some recommendations from the source guidelines. The use of HBA1c in screening for diabetes and FIT in screening for colorectal cancer were only weakly recommended, while an additional weak recommendation using VIA was developed to address the lack of facilities and skills in cervical cancer screening using pap smear.

### 4.1 Challenges and solutions

Throughout the process, the team encountered challenges and took some steps to overcome them.

Similar methodology with the source guideline to the GRADE Adolopment approach (e.g. using of GRADE and EtD) was often mentioned to have quickly enabled the process of evidence adaption from developers from Tunisia and the Middle East.^31^, ^34^ It was noted that the USPSTF did not use the GRADE methodology to assess the evidence and develop the recommendations and strength.^35^ For PHEX, the TWG had to reassess the evidence and develop GRADE tables. Despite that, the evidence was sufficient to construct the EtD framework.

Collaboration with the developers of the source guidelines has been often cited as main enablers in the successful implementation of the GRADE ADOLOPMENT.^31^, ^33^, ^34^ For example, Tunisian developers on breast cancer cited their major facilitators was their collaboration with the European Commission Initiative on Breast Cancer (ECIBC) who gave them full access of the latter's guideline source materials.^34^ Our team might have also benefited in consulting and collaborating with the source developers.^31^, ^33^

Similar to other developers, there was not enough contextual evidence to support the recommendations, especially when it comes to values, preferences, feasibility, and economics.^31^ For PHEX, stakeholders from various sectors were invited to participate, providing the panel with multiple perspectives and contextual insights. They shared their expertize, which helped in the EtD framework and final formulation of the guidelines. Effective facilitation during panel meetings was found to be crucial for the successful implementation of the GRADE Adolopment.

Lack of knowledge of the adaptation process was cited by the developers of the Mexican guideline on osteoporosis which have delayed their work.^36^ To overcome this challenge, several online orientation and training activities were conducted, and all team members were provided with instructions, resource materials and templates to assist them in their roles. The steering committee worked closely with the team and provided guidance throughout the process by maintaining close and constant communication.

Lack of sustainable funding, lack of multisectoral representation during the development process, resistance from stakeholder and insufficient manpower was a major obstacle in the guideline development using the GRADE Adolopment among low‐ to middle‐income countries.^36^, ^37^, ^38^ For example, the Pakistan Guideline on diabetes mellitus prioritized those questions needing an EtD because there were too many guideline questions with limited manpower. To sustain the pool of critical mass of CPG developers, continuing recruitment and training mechanism should be put in place. For PHEX, an informal community of evidence‐based health practitioners was established to become the pool of reviewers. Continuing capacity building activities via online and face to face were done to bolster the number of individuals with the skill to create guidelines.

Several developers utilized GRADEPro to coordinate with panelists during EtD development, which significantly aided them. GRADEPro allows creation of ED framework more convenient because of the set template and format.^31^, ^33^, ^34^ For PHEX, a manually created survey form had to be made. Moving forward, using the GRADEPro software, may lessen the amount of work in creating the EtD.

Implementing the project within the 6‐month timeline was found to be challenging and was similar to the experience of other developers.^31^ The team members dedicated more time to ensure that the guideline was completed within the timeframe which were also done by another developers.^31^ A timeline of 9 months for guideline development using GRADE Adolopment is sufficient for quality CPG development work. This includes a more well‐planned timeline considering planning, capacitating and recruitment.

## 5 CONCLUSION

Our experience has shown that the GRADE‐ADOLOPMENT framework is feasible and a valuable resource for developing CPGs in settings where resources are scarce. The framework was useful in incorporating Philippine‐specific contextual factors that differed from the primary source guideline, leading to necessary modifications. In addition, the GRADE‐ADOLOPMENT provided guidance throughout the course of the guideline development, despite the varied backgrounds, biases, and levels of experience from the technical team.

Using the Evidence‐to‐Decision framework allowed the panel to decide in a consistent and predictable manner. This process improved transparency, and highlighted the areas in which the consensus panel could adapt the guidelines to the Philippine context. The GRADE‐ADOLOPMENT process allowed the team to create de‐novo recommendations that simultaneously not only provided clinical guidance, but also transparency regarding the lack of information available.

Implementation research is currently being conducted to assess the acceptability, appropriateness and feasibility of the framework in the Philippines.

Despite all the challenges, the team was able to quickly and efficiently generate the needed guidelines through the GRADE‐ADOLOPMENT. Its use can be recommended into the guideline development methodology in the Philippines.

## AUTHOR CONTRIBUTIONS

Ian Theodore Cabaluna contributed to conceptualization, funding acquisition, investigation, methodology, project administration, original draft writing, and review and editing. Maria Vanessa Sulit supported conceptualization, investigation, methodology, and review and editing of the manuscript. Katelyn Edelwina Legaspi focused on investigation and original draft writing, while Leonila Dans provided conceptualization, supervision, funding acquisition, and review and editing contributions.

## CONFLICT OF INTEREST STATEMENT

The authors have no conflicts of interest to disclose.

## ETHICS STATEMENT

This article did not involve human participants in a research setting. It discussed the methodology for developing clinical practice guidelines, hence no ethics review was done.

## Supporting information

Supporting information.

## Data Availability

The data that supports the findings of this study are available in the supplementary material of this article.
