# Supplementary material for: Using the GRADE‐ADOLOPMENT framework in developing the Philippine national screening guideline recommendations
Source: Clin Public Health Guidel. 2024 Dec 17;2(1):e70008. doi: 10.1002/gin2.70008 (PMC13563408; doi:10.1002/gin2.70008)
Supplement: Supplementary file 1 [file GIN2-2-e70008-s001.docx]

Appendix 1. Gantt Chart of Project

Appendix 2. Recommendations of PHEX 2021 Phase 1 and Primary Source Guidelines

|  | **PHEX Clinical question** | **PHEX 2021 Phase 1 Recommendation** | **Primary Source guidelines recommendation** | **PHEX consensus panel considerations and differences from source guideline** |
| --- | --- | --- | --- | --- |
| 1 | Should asymptomatically apparently healthy adults be screened for lipid disorder? | Among asymptomatic apparently healthy adults aged 40-75 years with one or more CV risk factors, we suggest screening for lipid disorder using a lipid profile test. (conditional recommendation, low certainty evidence)  Among asymptomatic apparently healthy adults aged 18-39 years, we suggest against screening for lipid disorder (conditional recommendation, no evidence) - | **United States Preventive Services Task Force (USPSTF)** ^13^  Guideline recommends initiating use of low- to moderate- dose statins in adults aged 40-75 years without a history of CVD who have 1 or more CVD risk factors (dyslipidemia, diabetes, hypertension, smoking) and have a calculated 10-year risk of a cardiovascular event of 10% or greater**  Necessary to the calculation of the 10 year risk is a lipid screening test. (moderate certainty)  Recommends neither for nor against screening for dyslipidemia in adults aged 21-39 years old. Insufficient evidence. | The USPSTF guideline did not specifically focus on lipid screening, but rather on whether to start taking statins or not. The recommendation on lipid screening was based on the fact that it is necessary to conduct lipid profile testing to determine the 10-year cardiovascular risk.  Additionally, there was no strongly recommended frequency of screening for cardiovascular risk factors or lipid profiles. The USPSTF stated that screening every 5 years was reasonable, with increasing or decreasing frequency depending on the individual patient’s risk level.  Some PHEX panelists pointed out that there still needs to be further research evaluating the direct effect of screening for lipid disorders. |
| 2 | Should asymptomatic apparently healthy adults be screened for hypertension? | Among asymptomatic apparently healthy adults, we recommend screening for hypertension (strong recommendation, moderate certainty evidence). | **USPSTF** ^14^  Screen for hypertension in adults 18 years or older without known hypertension with office blood pressure measurement (OBPM). Blood pressure measurements should be obtained outside of the clinical setting for diagnostic confirmation before starting treatment.[^21^](https://www.zotero.org/google-docs/?jHuSzS) | No significant difference |
| 3 | Should asymptomatic apparently healthy adults be screened for type 2 diabetes mellitus in adults using hemoglobin A1c or fasting blood sugar? (FBS) | Among apparently healthy adults aged 40 years and above, or younger if with risk factors, we recommend screening for type 2 diabetes mellitus using fasting blood sugar (strong recommendation, moderate certainty evidence)  Amongst apparently healthy adults aged 40 years old and above, or younger if risk factors*, we suggest screening for type 2 diabetes mellitus using hemoglobin A1c (conditional recommendation, moderate certainty evidence). | **USPSTF**^15^  The USPSTF recommends screening for abnormal blood glucose as part of cardiovascular risk assessment in adults aged 40 to 70 years who are overweight or obese. Clinicians should offer or refer patients with abnormal blood glucose to intensive behavioral counseling interventions to promote a healthful diet and physical activity. (B recommendation) | The USPSTF lists HbA1c, fasting plasma glucose test, and oral glucose tolerance test without preference. Choice of screening test depends on the patient’s circumstance and clinician’s recommendation.  However, PHEX provided only a conditional recommendation for HBA1c due to contextual circumstances in the Philippines. This test is not readily available in the country, and has yet to be standardized between testing centers. Different laboratories may provide various ranges of reference values. |
| 4 | Should asymptomatic apparently healthy adults and children be screened for malnutrition using anthropometric measurements (e.g., BMI, MUAC, WHR) | Among asymptomatic apparently healthy adults, we recommend using body mass index in screening for obesity  (strong recommendation, moderate certainty evidence).  Among asymptomatic apparently healthy children (6-59 months), we suggest screening for acute malnutrition using mid-upper arm circumference or weight-for-height z-scores.  (conditional recommendation, low certainty evidence). | **Canadian Task Force on Preventive Health Care (CTFPHC)** ^16,17^  Recommendation for adults:  For apparently healthy adults ≥ 18 years old and above, they recommended measuring height, weight, and calculating BMI at the appropriate primary care visits (Strong recommendation; very low quality evidence)  Recommendation for children:  For all children aged 17 and younger  We recommend growth monitoring* at all appropriate primary care visits using the 2014 WHO Growth Charts for Canada.  Growth monitoring consists of measurement of height or length, weight, and BMI calculation or weight-for-length according to age. | It should be noted that for both children and adults, the focus of the CTFPHC was overnutrition and obesity. There was not much discussion on undernutrition or malnutrition, both of which are still prevalent problems in the Philippines.  Additionally, one difference from the source guideline is that the PHEX panel decided to use the Asia-Pacific Classification for cut-off scores on BMI as that was deemed more appropriate to the local setting. |
| 5 | Should hemoglobin and hematocrit be used in screening for anemia in apparently healthy adults? | Among asymptomatic apparently healthy non-pregnant adults, we suggest against routine screening of anemia using hemoglobin and/or hematocrit (conditional recommendation, very low certainty evidence). | No source CPGs were identified. | De-novo guideline creation. Most of the PHEX panel members favored no screening. However, this was given a conditional recommendation to account for patients at risk of occupational diseases. |
| 6 | Should serum creatinine and/or urinalysis be used to screen for chronic kidney disease among apparently healthy adults? | Among asymptomatic apparently healthy adults, we suggest against screening for chronic kidney disease using urinalysis (conditional recommendation, low certainty evidence). - de novo  Among asymptomatic apparently healthy adults, we recommend against screening for chronic kidney disease using serum creatinine (strong recommendation, no evidence). - de novo | No source CPGs were identified. | The consensus panel considered the following when formulating this recommendation:  ▪ Given the burden of the disease, chronic kidney disease should be prioritized.  ▪ Majority, however, agreed based on the net effects of the test that they do not favor screening. They acknowledged the low sensitivity values of urine dipstick and no diagnostic study to be used as evidence on serum creatinine. Moreover, even if these screening tests are not expensive and widely available, further work-up to ascertain the case of CKD and treatment of these cases will be costly. Accessibility to treatment services for CKD also remains a challenge. |
| 7 | Should chest x-ray be used to screen for lung cancer among apparently healthy adults? | Among asymptomatic apparently healthy adults, we suggest against screening for lung cancer using a chest X-ray (conditional recommendation, low certainty evidence). | **USPSTF**^18^  The USPSTF recommends annual screening for lung cancer with low-dose computed tomography (LDCT) in adults aged 55 to 80 years who have a 30 pack-year smoking history and currently smoke or have quit within the past 15 years. Screening should be discontinued once a person has not smoked for 15 years or develops a health problem that substantially limits life expectancy or the ability or willingness to have curative lung surgery. (B recommendation)  Chest radiography and sputum cytologic evaluation have not shown adequate sensitivity or specificity as screening tests. Therefore, LDCT is currently the only recommended screening test for lung cancer. | The consensus panel considered the following when formulating this recommendation.  ▪ Although lung cancer is a priority in screening apparently healthy adults, the majority agreed that the net effect of using chest X-ray for screening was not convincing.  ▪ Screening entails considerable cost for the confirmatory tests or the post-screening work-up. Furthermore, screening using chest X-ray is not cost-effective among the general or asymptomatic population.  ▪ The cost of these procedures, including the post-screening, is too expensive for marginalized sectors. Availability of chest X-ray in different parts of the country was also raised as a concern. |
| 8 | Should chest x-ray be used to screen for tuberculosis among apparently healthy adults? | Among asymptomatic apparently healthy adults, we suggest screening for pulmonary tuberculosis using a chest X-ray (conditional recommendation, very low certainty evidence). | WHO Consolidated Guidelines on Tuberculosis 2021 ^19^  1. Systematic screening for TB disease may be conducted among the general population in  areas with an estimated TB prevalence of 0.5% or higher  (updated recommendation: conditional recommendation, low certainty of evidence)  9. Among individuals aged 15 years and older in populations in which TB screening is recommended, systematic screening for TB disease may be conducted using a symptom screen, chest X-ray or molecular WHO-recommended rapid diagnostic tests, alone or in combination (new recommendation: conditional recommendation, very low certainty of evidence for test accuracy). | Given that the WHO lists CXR as a valid screening option for TB in high burden countries, there is no significant difference from the source guideline.  However, it should be highlighted that there was a debate among the panel members on whether to recommend CXR over other options such as sputum microscopy or rapid diagnostic tests alone or in combination. Ultimately, the recommendation was retained due to the hyperendemicity of TB in the Philippines. |
| 9 | Should electrocardiogram (ECG) be used to screen coronary artery disease among asymptomatic apparently healthy adults? | Among asymptomatic apparently healthy adults, we recommend against the use of resting or exercise ECG to screen for coronary artery disease (strong recommendation, low certainty evidence). | USPSTF ^20^  The USPSTF recommends against screening with resting or exercise electrocardiography (ECG) to prevent cardiovascular disease (CVD) events in asymptomatic adults at low risk of CVD events.  Screening with exercise ECG does not improve health outcomes , even in higher risk populations (those with diabetes mellitus) | No significant difference |
| 10 | Should visual inspection with acetic acid (VIA) followed by colposcopy or pap smear followed by colposcopy be used to screen for cervical cancer in asymptomatic adult women? | 1. Among asymptomatic women aged 21 to 65, we recommend screening for cervical cancer every three years using a Pap smear (strong recommendation, moderate certainty evidence)  2. Among asymptomatic women aged 21 to 65, we recommend screening for cervical cancer every three years using visual inspection with acetic acid as an alternative to Pap smear  (strong recommendation, moderate certainty evidence) | USPSTF^21^  The USPSTF recommends screening for cervical cancer every 3 years with cervical cytology alone in women aged 21 to 29 years. For women aged 30 to 65 years, the USPSTF recommends screening every 3 years with cervical cytology alone, every 5 years with high-risk human papillomavirus (hrHPV) testing alone, or every 5 years with hrHPV testing in combination with cytology (cotesting). | The USPSTF recommends cervical cytology and HPV testing. VIA is not mentioned.  However, the PHEX panel still decided to recommend visual inspection with acetic acid as an alternative to pap smear despite the moderate certainty of evidence due to issues of inequity and accessibility. Much of health care expenditure in the Philippines is still out of pocket. VIA is much more accessible in terms of cost and in terms of laboratory infrastructure than Pap smears of other forms of cervical screening. Many areas in the country do not have the means or infrastructure to carry out the confirmatory testing and treatment that would be required if other screening methods are used. Additionally, the VIA test is a method widely used in this country, and many practitioners are comfortable administering it.  Panelists also agreed not to place a specific recommendation on using these screening tools followed by colposcopy since not all abnormalities found in Pap smears or VIA will lead to testing using colposcopy. |
| 11 | Should fecal occult blood test followed by colonoscopy be used to screen for colorectal cancer in asymptomatic apparently healthy adults? | Among asymptomatic apparently healthy adults aged at least 50, we recommend to screen for colorectal cancer using annual FOBT or FIT, followed by colonoscopy, when indicated.  (strong recommendation, high certainty evidence) | **USPSTF**^22^  Screen all adults aged 45 to 75 years old for colorectal cancer. Recommended screening strategies include   - High-sensitivity guaiac fecal occult blood test (HSgFOBT) ot fecal immunochemical test (FIT) every year - Stool DNA-FIT every 1-4 years - Computed tomography colonography every 5 years - Flexible sigmoidoscopy every 5 years - Flexible sigmoidoscopy every 10 years + annual FIT - Colonoscopy screening every 10 years   Selectively screen adults aged 76-85 years for colorectal cancer | The USPSTF guideline recommends tests that are not yet widely available or affordable in the Philippines.  Additionally, colonoscopy is recommended at regular intervals by the USPSTF, while for PHEX it is only performed when indicated. This is due to the prohibitively high cost of colonoscopy in the Philippines. Although many practitioners are trained to perform it, its accessibility is limited, especially in rural areas.  Additionally, age at initiation and termination of screening is different for the two guidelines. |
| 12 | Should asymptomatic apparently healthy adolescents and adults be screened for unhealthy alcohol use? | Among asymptomatic apparently healthy adults, we recommend screening for unhealthy alcohol use and providing persons identified with risky drinking with brief behavioral counseling intervention.  (strong recommendation, moderate certainty evidence)  Among asymptomatic apparently healthy adolescents, we suggest screening for unhealthy alcohol use and providing persons identified with risky drinking with brief behavioral counseling intervention (conditional recommendation, low certainty evidence). | **USPSTF^23^**  USPSTF recommended that clinicians screen adults 18 years or older for alcohol misuse and provide brief behavioral counseling interventions to those engaged in risky or hazardous drinking behaviors (B recommendation).  They also concluded that the evidence in adolescents was insufficient to evaluate the balance of benefits and harms of screening and behavioral counseling interventions to reduce alcohol misuse (I statement) [^25,26^](https://www.zotero.org/google-docs/?BZsqmQ) | The USPSTF categorized their recommendation regarding adolescents as an I statement. This means that they found the evidence insufficient, and declined to provide a recommendation.  PHEX decided to give an outright recommendation regarding screening despite the low certainty of evidence. The panel agreed that although the effectiveness of a particular screening tool depends on the age group being studied, their use was still perceived to be equitable, acceptable, and feasible. They also decided to proceed with the recommendation because unhealthy alcohol use was deemed a priority risk factor to screen, where benefits of screening outweigh undesirable effects. |
| 13 | Should asymptomatic apparently healthy adolescents and adults be screened for high risk sexual behavior? | Among asymptomatic apparently healthy adolescents and adults, we suggest screening for high-risk sexual behavior (conditional recommendation, low certainty evidence). | No source CPGs were identified. | Considerations  The consensus panel considered the following when formulating this recommendation:  ▪ Most agree that high-risk sexual behavior is a priority mainly because of the rising cases of HIV.  ▪ All agree that screening has benefits that outweigh the harmful effects (i.e., stigma) provided that health workers that will be involved are well-trained in handling those who will be identified.  ▪ The CP perceived the screening to be inexpensive. However, professional fees and the battery of tests might incur high costs.  ▪ All agree that screening for high-risk sexual behavior using suggested tools is equitable, acceptable, and feasible.  ▪ Issues of privacy and confidentiality, labeling, stigma, and acceptance of the result were raised.  This recommendation reached consensus. However, there was an abstention from the panel due to the concerns about privacy and legal issues on screening minors. |
| 14 | Should apparently healthy adults (excluding pregnant women) and adolescents be screened for tobacco smoking? | Among all adults, we recommend that healthcare providers screen for tobacco smoking (strong recommendation, low certainty evidence).  Among all adolescents, we recommend that healthcare providers screen for tobacco smoking (strong recommendation, very low certainty evidence). | **USPSTF**^24^  The USPSTF recommends that clinicians ask all adults about tobacco use, advise them to stop using tobacco, and provide behavioral interventions and U.S. Food and Drug Administration–approved pharmacotherapy for cessation to adults who use tobacco. (A recommendation)  The USPSTF recommends that clinicians ask all pregnant women about tobacco use, advise them to stop using tobacco, and provide behavioral interventions for cessation to pregnant women who use tobacco. (A recommendation)[^27^](https://www.zotero.org/google-docs/?jHqqOj)  The USPSTF recommends that primary care clinicians provide interventions, including education or brief counseling, to prevent initiation of tobacco use among school-aged children and adolescents (B recommendation). Electronic cigarettes are included in this recommendation.[^28^](https://www.zotero.org/google-docs/?rblyfJ) | Note that the USPSTF labeled its recommendations as category A or B, implying moderate to strong evidence. However, the PHEX guideline ranked the evidence as only low certainty. The difference in the recommendation stems from the fact that the PHEX panel limited themselves to screening for tobacco smoking, but the USPSTF already includes interventions within its guideline. |
| 15 | Should asymptomatic apparently healthy adolescents and adults be screened for depression? | Among asymptomatic apparently healthy adolescents and adults, we suggest screening for depression using PHQ-9 (conditional recommendation, low certainty evidence). | **USPSTF**^25,26^  The USPSTF recommends screening for depression in the general adult population, including pregnant and postpartum women. Screening should be implemented with adequate systems in place to ensure accurate diagnosis, effective treatment, and appropriate follow up.  The USPSTF recommends screening for major depressive disorder (MDD) in adolescents aged 12-18 years. | Similar recommendations, only difference is that PHEX named a specific questionnaire in the guideline itself. The USPSTF does list the PHQ-9 in its discussion of the recommendations, but other screening tests are mentioned as well. PHEX panelists recognized the accuracy of the Patient Health Questionnaire PHQ-9 and were uncertain in PHQ-2 in terms of diagnostic performance. |
| 16 | Should developmental screening tools be used in screening developmental delay in children aged 0-4? | Among asymptomatic apparently healthy children, we recommend against screening of developmental delay using developmental delay screening tools (strong recommendation, low certainty evidence). | **CTFPH** ^27^  We recommend against screening for developmental delay using standardized tools in children aged one to four years with no apparent signs of developmental delay and whose parents and clinicians have no concerns about development (strong recommendation; low quality evidence) | No significant change when compared to the Canadian Task Force guideline. However there were differences in the recommendations and context when compared to other guidelines such as the USPSTF[^32^](https://www.zotero.org/google-docs/?No55Qh) and American Academy of Pediatrics[^33^](https://www.zotero.org/google-docs/?KWBs3N)  The majority of the panel agreed that screening leading to early intervention was a desirable effect, however they were undecided on how to mitigate the issue of undesirable effects of screening tests.  PHEX also placed a caveat that although the use of standardized questionnaires cannot be recommended at this time, practitioners are not discouraged from checking for delays in development during routine checkups through the caregivers’ reports and child’s developmental milestones. |
